# Exposure to subtle dominance cues activates the stress response and affects decision-making

**DOI:** 10.3389/fpsyg.2025.1517308

**Published:** 2025-06-26

**Authors:** Maryam Bamshad, Karina Xie, Rema Rasheed, Kathryn Holt, Grace Assabil-Bentum, Nicholas B. Aoki

**Affiliations:** ^1^Department of Biological Sciences, Lehman College, CUNY, Bronx, NY, United States; ^2^Department of Linguistics, University of California Davis, Davis, CA, United States

**Keywords:** risk assessment, skin conductance response, SCR, arousal, condescension, anger, trust

## Abstract

**Introduction:**

Dominance cues may offend and elicit anger. Based on theories of affect-as-information, we tested whether subtle cues in words or images indicative of dominance could activate the stress response and impact decision-making.

**Methods:**

Participants asked to imagine being patients were exposed to subtle dominance cues of a doctor. By measuring the skin conductance levels and through self-reported assessments, we examined whether participants would be offended when exposed to dominance cues in text alone or when combined with facial images. Participants assessed the probability of a medication’s side effects and chose to take the medication prescribed after reading a doctor’s advice that was worded to sound either condescending or neutral. The doctor’s statements were shown alone or matched with a photo of either a dominant-looking or a trustworthy-looking face.

**Results:**

Most found dominance cues presented in text, with or without a facial image, offensive. No differences were found in probability assessment but the choice to take the medication was affected when the condescendingly worded text was combined with a photo of a dominant face. Arousal levels increased while viewing the dominance cues, but the levels decreased when decisions were made following exposure to a condescendingly worded text and its matching facial expression of dominance.

**Discussion:**

The study contributes to understanding the impact of lower-intensity emotions sensed during social interactions on decision-making, which could be important for designing computer programs that mimic human social interactions.

## 1 Introduction

Anger is a negative emotion that is known to affect decision making. Evoking the emotion by exposing participants to highly aversive texts, images, or sounds decreases people’s risk estimates of events and influences their decisions (Lerner and Keltner, 2000; Habib et al., 2015; Lerner et al., 2015). Although anger can be stimulated by aversive cues, it could also be elicited by more subtle stimuli such as spoken or written words that are perceived as disrespectful (Miller, 2001). Condescending language is an example of a subtle form of aggression, regarded as disrespectful (Ortiz, 2022; Perez-Almendros and Schockaert, 2022). The language is considered as implicitly abusive because it does not involve profanity, yet the word choices and speech pattern signify a difference in status between a speaker who assumes superiority versus the listener (Perez-Almendros and Schockaert, 2022). Datasets derived from online sources such as Reddit have been used to show that a condescending manner of speech, signifying superiority, can be detected from the addition of single words to a written dialog (Wang and Potts, 2019). These studies indicate that experts may be well-intentioned and respectful, but their statements could be construed as condescending by the reader (Wang and Potts, 2019).

Superiority can be conveyed not only through condescending words but also via facial gestures that are indicative of a dominant status (Mignault and Chaudhuri, 2003). People are sensitive to slight changes in facial expressions. When presented with unfamiliar faces, they quickly form an impression of the targets’ dominance or trustworthiness from a hint of anger or happiness, respectively. These trait perceptions can have consequences for decision making (Todorov et al., 2015; Li et al., 2022). For example, people choose those they perceive as dominant to lead them in context of a wartime scenario but those they perceive as trustworthy to lead them in context of a peacetime scenario (Ferguson et al., 2019). Also, college-age students tested for their trust in others tend to invest more money with adults whose faces are significantly older than themselves and are considered unattractive compared to younger adult faces (Li et al., 2022). Research has shown that these impressions are linked to emotions. For instance, cues that express dominance are related to anger and can affect people’s behavior (Cabral et al., 2016). Respondents are reported to speak less and agree more with doctors that exhibit high dominance compared to those that exhibit low dominance or trustworthiness (Schmid Mast et al., 2008).

To stimulate emotions, studies often use aversive images (Gerdes et al., 2010). Although these cues can elicit strong arousal by prompting participants to visualize an unpleasant scene, they may not be realistic scenarios for decision making during social encounters where emotions could be implicitly expressed. For example, in business or healthcare work settings, and in interpersonal relationships, people who exert dominance can elicit anger by using condescending statements when talking to others (Canary et al., 1998; Manderino and Berkey, 1997; Raghid and Vongas, 2022). The recipients of such reactions are sensitive to small variations in facial expression or words, (Planalp, 1996; Schmidt and Cohn, 2001; Frith, 2009), so it should be possible to arouse their negative emotions by exposing them to these subtle cues. However, less is known about the arousal’s effect elicited by low intensity emotional stimuli during social interactions (Frijda et al., 1992) and their impact on decision making.

According to the affect-as-information model, negative or positive emotions provide information about the value and significance of an event and guide thoughts in choosing an option or making a decision (Clore, 1992; Schwarz, 2012). When emotion-evoking cues are sensed by the body, the information reaching the brain focuses the mind on important events, aid us in making comparisons, and motivate us to act so we can make choices or decisions (Peters et al., 2006). This type of information processing is thought to be mediated by a physiological response known as stress (Phelps et al., 2014). When making choices, emotions sensed through the body guide the brain in entering a hypervigilant state and thus focus our attention on incoming information. This response is achieved partly through the activation of the sympathetic nervous system (SNS) via a pathway within the brain that ends in locus coeruleus of the brainstem causing arousal (Peters et al., 2017).

Emotion-stimulated arousal is commonly tested with biometric tools that provide a consistent measure of the skin conductance response (SCR) (Bradley, 2000; Dawson et al., 2016). The SCR frequency and amplitude reflect the activity of sudoriferous sweat glands that are controlled by the sympathetic nervous system. Hence, SCR is considered as an indirect measure of the stress response (Christopoulos et al., 2016). Different emotions such as anger, fear, disgust, surprise, joy, and sadness elicited by exposure to sensory cues can be distinguished based on the resulting pattern of an individual’s SCR activity (Collet et al., 1997). A direct relationship found between the SCR strength and participants’ self-reported degree of arousal to the emotion-evoking cues has reinforced the reliability of the technique (Lang et al., 1993). In decision-making studies, SCR has been used to show how exposure to distress-stimulating cues results in arousal that focuses attention on the most important information and thus alters participants’ decision strategies (Wichary et al., 2016).

The autonomic stress response may be stimulated differently by faces and words that express emotions. Using SCR, a recent study examined arousal elicited by facial images versus words that expressed the same emotions or were neutral. The same stimuli were presented in multiple trials for various emotions such as anger, fear, disgust, surprise, sadness, and happiness to determine if differences exist in arousal for faces versus words and if arousal levels decrease due to habituation following repeated exposure to the same stimuli. Results showed that at initial exposure, arousal levels were higher for faces than for words for all emotions except for fear and anger, and the pattern of habituation differed for various emotions (Juuse et al., 2024). Studies using techniques other than SCR have corroborated that words and faces with the same emotional meaning are processed differently in the brain (Ballotta et al., 2023; Bayer and Schacht, 2014) presumably because facial emotion processing for social interactions evolved earlier than spoken words (Rellecke et al., 2011).

To our knowledge, there is little work that has examined whether subtle dominance or trustworthy cues depicted through a combination of words and facial expressions can affect a person’s preferences for the producer and alter the decision-making of that person by impacting their autonomic stress responses. We designed the current study to test whether neutral words added to written statements would be perceived as condescending when viewed together with a dominant or a trustworthy facial image. Based on linguistics research, we created scenarios to expose participants to condescending language. Research has shown that participants can detect subtle variations in text as condescending. These statements do not have to be explicitly negative or critical. By inserting assertive words like “must” or “should,” a statement can be altered to be perceived as condescending. However, these words must be included within a dialog rather than expressed as isolated words within a statement and occur within a social context (Wang and Potts, 2019).

To create ecologically valid scenarios for social interactions, we chose the doctor’s office as a setting. When talking to patients, some doctors exhibit dominance, which can be expressed explicitly through their speech or implicitly through nonverbal gestures, such as changes in facial expression or tone of voice (Ambady et al., 2002a, b; Mast, 2007; Pilnick and Dingwall, 2011). Researchers have investigated the effect of the doctor’s communicative style on the patients’ satisfaction, preferences, and compliance with mixed results. Some have reported lower satisfaction and preferences for doctors who exhibit dominance (Roter et al., 1997; Ambady et al., 2002b), whereas others have found variability in satisfaction and compliance with prescribed advice based on the patient’s characteristics and the doctor’s gender (Savage and Armstrong, 1990; Bradley et al., 2001). We wrote dialogs for doctors that read as condescending with or without corresponding facial expressions to create three conditions: (1) Dominant (dominant facial image with and without condescending remarks), (2) Trustworthy (trustworthy facial image with and without condescending remarks), (3) NoFace (no facial image with and without condescending remarks).

Based on the affect-as-information model (Clore, 1992; Schwarz, 2012), we assumed that exposure to subtle dominance cues would stimulate anger to impact preferences for the doctor, risk comprehension for drug side effects, and decisions to take medications. To investigate how words and facial expressions may differentially alter arousal levels based on the participant’s perceptions of the speaker, we conducted a within-subjects experiment. Additionally, we studied changes in arousal levels to determine if they would habituate with repeated exposure to similar stimuli as previously shown (Juuse et al., 2024). We tested the following four hypotheses:

*H1*: The addition of neutral words such as “simple” and “obviously” to statements of advice given by a doctor to a patient would be perceived as condescending.

*H2*: Condescending remarks would reduce the participant’s preference for the doctor as shown by the choice to travel less time and pay less to visit the doctor.

*H3*: Anger in reaction to words and faces that match in conveying dominance would lower the estimate for the medication side effects and decrease the decision to take the medication prescribed by the doctor.

*H4*: Arousal levels assessed by SCR levels would increase when words and faces match in conveying dominance when viewing dialogs and making decisions and decrease as participants habituate to the presented scenarios in multiple trials.

## 2 Materials and methods

### 2.1 Materials

#### 2.1.1 Participants

A total of 200 college students enrolled in introductory biology courses were invited to participate in the study for course credit. The college, located in Bronx NY, serves a diverse population of students. The majority are female minorities with an average household income below $30,000. Of the invited students, 140 came to the lab to serve as participants. The consent form they signed and the experimental procedures were approved by the Institutional Review Board (IRB) at Lehman College, CUNY.

#### 2.1.2 Procedure

Using LimeSurvey, we designed two surveys. The first consisted of hypothetical medical scenarios and the second included demographic questions. The participants completed the medical-scenarios survey while their arousal levels were assessed with a biometric sensor. Upon completion, the sensor was removed, and the participants were directed to complete the demographics survey.

#### 2.1.3 Medical-scenarios survey

**Figure 1:**
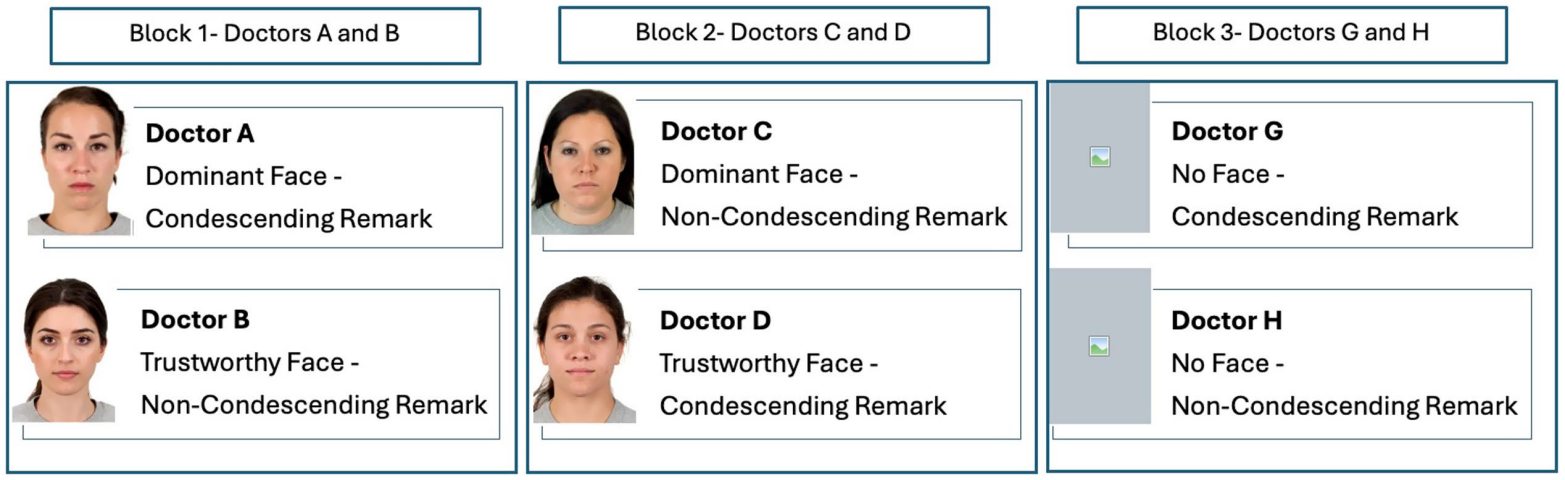
The stimulus was presented in three blocks. All participants viewed the three blocks in a counterbalanced order. In each block, a pair of doctors offered advice for a specific disorder and prescribed medication to treat that disorder. In blocks 1 and 2, the doctor’s remarks were accompanied by the doctor’s image. In block 3, the doctor’s remarks were shown without an image. In block 1, the doctor’s facial expression and words were matched for emotions (dominant/condescending, trustworthy/non-condescending). In block 2, the doctor’s facial expression and words were unmatched (dominant/non-condescending, trustworthy/ condescending). Facial images reproduced with permission from Ma et al. (2015).

For the medical scenarios, we created three blocks of doctor-patient dialogs. In each block, a pair of doctors identified with alphabet letters (Doctors: A and B, C and D, G and H), gave medical advice for one of three different disorders. Within each block, statements were written for each pair of doctors to advise treating one of three disorders. The advice in block one was to lower cholesterol levels, in block two to lower blood sugar levels, and in block three to lower blood pressure. We selected disorders that are listed as top risks for morbidity and mortality worldwide (World Health Organization, 2016). For two pairs of doctors (A/B, C/D), we added an image of a face representing the doctor. For the other pair of doctors (G/H), no facial images were shown (Figure 1). The facial images were selected from the Chicago Face Database, version 3.0 (Ma et al., 2015) whose participants were not involved in the current study. These images were collected based on prior norming studies. The database consists of high-resolution, standardized photographs of male and female faces of varying ethnicity between the ages of 17-65 years accompanied by norming data. The pictures of faces were previously rated by participants for a variety of attributes including emotions. From the available list, we selected four images of white females ages 23–28. Based on the Chicago Face Database, two of the images were rated high for dominance (mean = 4.19, 3.72) and lower for trustworthiness (mean = 3.09, 3.11); the other two images were rated high for trustworthiness (mean = 4.52, 4.21) and lower for dominance (mean = 2.22, 2.15). Previous studies have shown that sex, age, and ethnicity of the person depicted in a facial image can influence whether they are perceived as dominant, affiliative, and trustworthy (Hess et al., 2000; Li et al., 2022). As the population from which we pool our participants are mostly young females, we controlled for the effects of age and sex by using images of young females to represent the doctor so that their attributes would align with that of the participant. We used white females to represent the doctor’s ethnicity because we could not control for ethnicity given the diverse ethnic background of our participant pool.

**Figure 2:**
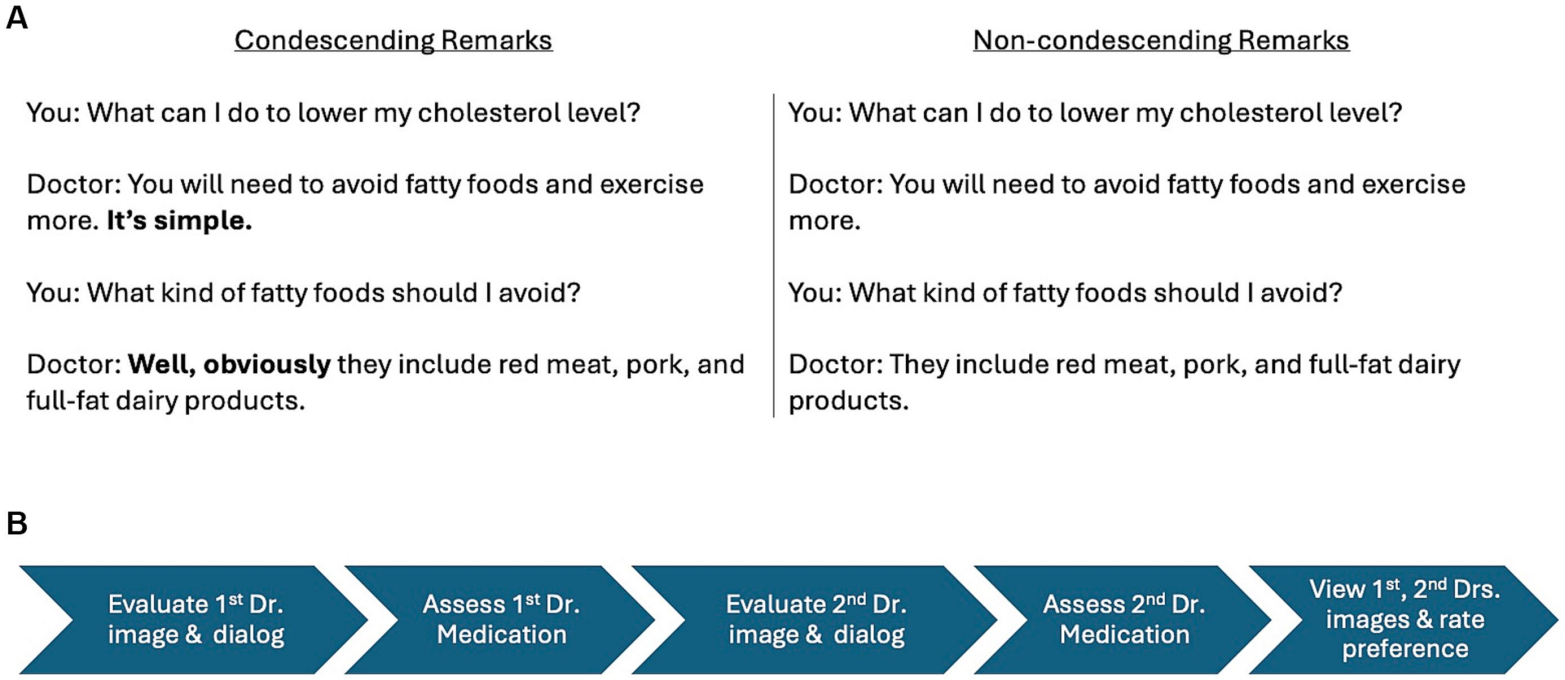
Prototype of a doctor-patient dialog. Within each block, one doctor offered advice for a specific disorder using condescending remarks, whereas the other doctor offered the same advice using non-condescending remarks **(A)**. Timeline of stimulus presentation for the two doctors within each block **(B)**.

The statements created for each pair of doctors in each block had the same syntax and were presented in the same format as a dialog between a doctor and a patient. Based on previous research on detecting condescension (Wang and Potts, 2019), we created a dialog between the patient and the doctor. The dialog consisted of two statements made by each doctor in response to two questions asked by the patient. One doctor in each pair made condescending remarks, whereas the other doctor made non-condescending remarks. To create condescending remarks, we inserted the word “simple” in the first statement and “obviously” in the second statement. To create non-condescending remarks, we omitted those words. Thus, one doctor in each pair within each block was made to sound condescending and the other to sound non-condescending. For one pair of doctors (A and B) in block one, the text’s language and the facial cues were congruent such that the doctor making the condescending remarks looked dominant and the doctor making the non-condescending remarks looked trustworthy. For the other pair of doctors (C and D) in block two, the text’s language and facial cues were noncongruent such that the doctor making the condescending remarks looked trustworthy and the doctor making the non-condescending remarks looked dominant (Figure 2A).

In the hypothetical scenario, we asked participants to imagine that each doctor they visited had prescribed them a medication with some adverse events. For each medication, participants viewed a table with a list of possible side effects shown with risk labels (“Very Common,” “Common,” “Uncommon,” “Rare,” and “Very Rare”). The listed adverse events for each medication were presented in the same verbal format but the adverse effects were slightly modified to create variation of a similar severity level. For example, “very common” and “very rare” side effects of the medication to treat high sugar levels were “diarrhea” and “life-threatening lactic acidosis” respectively and to treat blood pressure they were “dehydration” and “life-threatening rash” respectively.

Having viewed the tabular list of side effects, the participant was asked to estimate the probability (in percentages from 0 to 100) of being afflicted with the “Uncommon” side effect should they take the medication. For each medication, we asked two follow-up questions: (1) indicate on a 5-point Likert scale (Very unlikely to Very likely), the likelihood that you would take the medication, and (2) select from a list of options the reason why you would or would not take the prescribed medication. The options given were a mix of positively and negatively worded statements such as: (a) most of the adverse events are very unlikely, (b) A lot of people will experience at least one of the adverse events, (c) I do not trust doctors, (e) the rare side effect shown is a very serious condition. We adapted this scheme to test the participants’ probability estimates and decisions to take medications from a previous study that studied the overestimation effect using Consumer-Medication-Information (CMI)-like list available from a pharmacy and the Physicians’ Desk (Peters et al., 2014).

#### 2.1.4 Demographics survey

The demographics survey was administered on LimeSurvey separately and immediately after the medical-scenarios survey to avoid participant fatigue. In the demographics survey, participants answered questions about their age, gender, ethnicity, whether they were the first in their family to go to college, and whether one or both of their parents had a college degree. They were also asked if they had any previous negative experiences with doctors. Two questions were added to determine their understanding of the linguistic and mathematical terms used in the survey: (1) explain the meaning of “condescending” in your own words; (2) if a medication you are asked to take has a side effect that is listed as “Uncommon,” what is your estimate of the probability (in percentage from 0 to 100) that you will experience that side effect by taking that medication. Lastly, we asked if participants were taking medications to treat any of the three disorders mentioned in the scenarios.

### 2.2 Methods

We used a within-subject experimental paradigm to expose each participant to the three blocks of doctor-patient scenarios in a counterbalanced order. Each participant saw the three pairs of doctors in a different order. The timeline for stimulus presentation within each block is shown in Figure 2B. Participants viewed the scenario in the following order: (1) the doctor-patient dialog and any corresponding image of the first doctor, (2) answered questions to evaluate the first doctor’s attitude, (3) decided on the medication prescribed by the first doctor, (4) viewed the doctor-patient dialog and any corresponding image of the second doctor, (5) answered questions to evaluate the second doctor’s attitude, (6) decided on the medication prescribed by the second doctor, (7) viewed the images and dialogs of both doctors again before answering questions to indicate their preference for the first and the second doctor. Although the order of the scenario’s presentation within each block was fixed, participants were allowed to move through the questionnaire at their own pace to estimate the medication side effects and make a decision, which followed each doctor-patient dialog presentation. This was to minimize the stress of being timed while estimating the medication’s adverse events and deciding whether to take the drug or not.

Seated in front of a laptop computer, the participant was instructed to imagine being diagnosed with a specific disorder and having to see a doctor for treatment. For each disorder, the participant was told to seek advice from a doctor and then ask for a second opinion from another doctor. Participants were then given a few questions to indicate their perception of the doctor and preference for each doctor. On a slider set to move from 0 to 10, participants rated their perception of the doctor, first for condescending attitude (0 = doctor was condescending to 10 = doctor was respectful) then for trustworthiness (0 = I would not trust the doctor at all to 10 = I would trust the doctor a lot). Above each measuring slider, we inserted an affective slider with emoticons showing an unhappy face and a happy face affective states at the two ends of the slider. The unhappy face was placed at 0 (condescending/I would not trust the doctor at all) and the happy face was placed at 10 (respectful/I would trust the doctor a lot). Both the measuring and the affective sliders had a horizontal orientation with the square-shaped thumb starting in the center of the slider. The emoticons of a happy face representing like (respectful/trust) and an unhappy face representing dislike (condescending/would not trust) were shown in addition to the numerical values to help the participants choose the emotion that best corresponded to their opinion of the doctor. We used the affective slider based on research showing that it is an effective self-reporting tool for assessing emotions (Betella and Verschure, 2016). After viewing the information provided by both doctors, we asked participants to rate their preference for each doctor by answering the following three questions: (1) which doctor do you prefer (on two scale points: doctor A or doctor B), (2) how much would you pay to see each doctor (on four scale points: I would not go to the doctor, less than $100, $100 to $300, $300 or more), (3) how many hours would you travel to see the doctor (on five scale points: 0 min to more than 2 h).

#### 2.2.1 Physiological responses

In addition to the self-reported assessments, we used biometric tools to acquire an objective measure of the physiological arousal of the participants while they assessed each dialog and each prescribed medication. To determine how the participants’ stress response would be activated, we recorded the skin’s conductance responses (SCRs) as a measure of the sympathetic nervous system activity during two phases while the participants were: (1) making an intuitive assessment of the doctor’s attitude by viewing the dialog, (2) deliberating to decide on the probability of side effects and whether to accept the prescribed medication. Upon their arrival at the biometric lab, participants were asked to complete a mock survey that was created for practice showing how to view images and texts within the actual survey. Each participant was prepared for the test by wearing a disconnected biometric sensor on the wrist to simulate the testing condition while an experimenter described the procedure. Participants were told to relax, breathe normally, follow all instructions, and pay attention. They were informed that their answers would not be judged, but their attention would be checked with a question randomly inserted into the survey.

After reading the instructions on the mock survey, they signed the consent form and followed the experimenter into a quiet and temperature-controlled testing room with adequate lighting and comfortable seating. Each participant was seated behind a laptop computer showing an oversized (+) sign on the screen and was given a headset to wear. The experimenter fitted a Shimmer3 GSR + (Galvanic Skin Response) device on the participant’s non-dominant wrist and attached it to two EDA (electrodermal activity) electrodes adhered to the palm of the hand. A partition separated the participant’s laptop from the experimenter’s computer equipped with the iMotions version 10.0 (Boston, MA) software. Once the baseline measurements were taken with the oversized (+) sign in view for 60 s, the participant was given access to the survey which took 25–30 min to complete. Immediately after testing, the GSR device and headset were removed, and the participant was directed to a second laptop to complete the demographics survey.

iMotions is a software designed to facilitate easy calibration, data acquisition, and analysis. The data are automatically synchronized and can be later annotated to collect SCR measurements for selected segments of the survey in response to stimuli of interest. To assess arousal, we used the frequency of EDA peaks (sudden shifts between the baseline tonic component and the reactive phasic component of EDA activity) and the duration and amplitude of each peak (μS) in response to specific stimuli that are provided by iMotions (Lei et al., 2017). iMotions also shows the signal quality and the duration of time (ms) spent viewing the stimuli, which we also collected for each participant.

#### 2.2.2 Statistical analysis

The survey responses and SCR recordings from iMotions were collated in Excel and analyzed in R (R Core Team, 2021, version 4.4.1). Before analysis, the survey questions that were categorical and on the Likert scale were converted to numbers. The SCR recordings for each participant were marked at two specific segments of the survey: (1) Dialog, and (2) Decision. The Dialog segment was when participants viewed the statements and images and indicated their perception of the doctor. The Decision segment was when participants viewed the medication’s adverse events, assessed the probability of the “Uncommon” side effect, and decided to take the medication or not. With the recordings thus annotated, iMotions calculated the signal quality for each participant, the peak frequency and peak amplitude, and the duration of time for the Dialog and the Decision segments.

The data were analyzed using Bayesian mixed-effect models fitted with the *brms* package (Bürkner, 2017) and *Stan* (Stan Development Team, 2023). We used Bayesian analysis rather than ANOVA because of its suitability for modeling the categorical data that we had collected for perception and preference ratings (Jaeger, 2008), and for its ability to accommodate the random effects structure of the study (Barr et al., 2013; Vasishth et al., 2018). The default weak priors from *brms* were employed. Two types of models were fitted: linear regression models (for numeric response variables) and ordinal regression models (for categorical response variables). Fixed effects for all models were categorical and sum-coded, including Statement, Face, and their interaction. The maximal random effects structure was employed (i.e., random intercepts for Participant, and by-Participant random slopes for Statement and Face). The R syntax of the model structure is shown in Equation 1 for clarity, where “X” refers to any one of the predictor variables in the current study.


(1) $$X\sim {\text{Statement}}^{*}\text{Face}+(1+\text{Statement}+\text{Face}\;|\;\text{Participant})$$

## 3 Results

The demographic survey data showed that the participants were 85% female with a mean/median age = 23.5/21. Most responded that they were not the first in their family to go to college (69%) but indicated that their parents do not have a college degree (63%). They identified their ethnicity as follows: 22.5% African/African American, 13.8% North American or Caribbean Latinx, 11.6% Afro-Caribbean or Afro-Latinx, 7.2% Pacific Islander/South Asian, 6.5% Middle Eastern, 5.8% White, 5.1% Central or South American Latinx, 3.6% East Asian/South East Asian, 3.6% Hispanic/Latino, 3.6% Indian/other nation in the Indian subcontinent, 3.6% preferred not to say, 2% other (North African, Indo-Caribbean, Indo-Guyanese), and 10.9% selected multiple categories such as African/African American/White, Central or South American Latinx/Native American/Alaskan Native.

When asked to describe what condescending means, 75% defined it as rude or gave a dictionary definition of someone acting superior to belittle the other. The remaining 25% were unable to accurately define the word, with 14% considering the term as something negative, 10% as something positive, and 1% saying they did not know. Asked if they ever had a negative experience with a doctor, 57% responded No and 43% responded Yes. Asked if they regularly took medications to treat disorders mentioned in the scenarios, 93% responded No for high cholesterol and blood pressure and 94% responded No for high blood sugar levels.

The medical scenario survey data for a total of 138 participants were analyzed. Two participants were removed from the final analysis because the SCR had failed to record during testing. Out of the 138 participants, we analyzed the SCR data for 39 who showed a peak in all the annotated survey segments. The data for the remaining 99 participants were not included in the analysis because they either showed no peaks or only a few peaks in the designated segments despite having an acceptable signal quality. Research has shown that individual differences in SCR signal are to be expected. In within-subject experiments, whereby all participants are exposed to the same stimuli, some show a large emotional response, but others show small or no response at all (Boucsein et al., 2012). Although alternative techniques are available that are less sensitive to peak detection thresholds (Benedek and Kaernbach, 2010), we chose to focus our analysis only on respondents that had detectable single distinct SCRs because we were interested in examining only those with the most robust emotional responses to subtle cues. Though the presence of non-responders in the present study resulted in removal of a large of number of respondents from the SCR analysis, we considered the remaining number of participants to be sufficient for statistical analysis based on previous studies using a range of 20 to 40 individuals for SCR testing in within-subject experiments (Banks et al., 2012; FeldmanHall et al., 2016). We conducted a sensitivity power analysis with G*Power version 3.1.9.6. It showed that a linear multiple regression with 2 predictors (facial expression and statement type) and 39 participants would be sensitive to effect of *r*^2^ = 0.3 with 80% power (alpha = 0.05).

### 3.1 Preferences and perceptions

**Figure 3:**
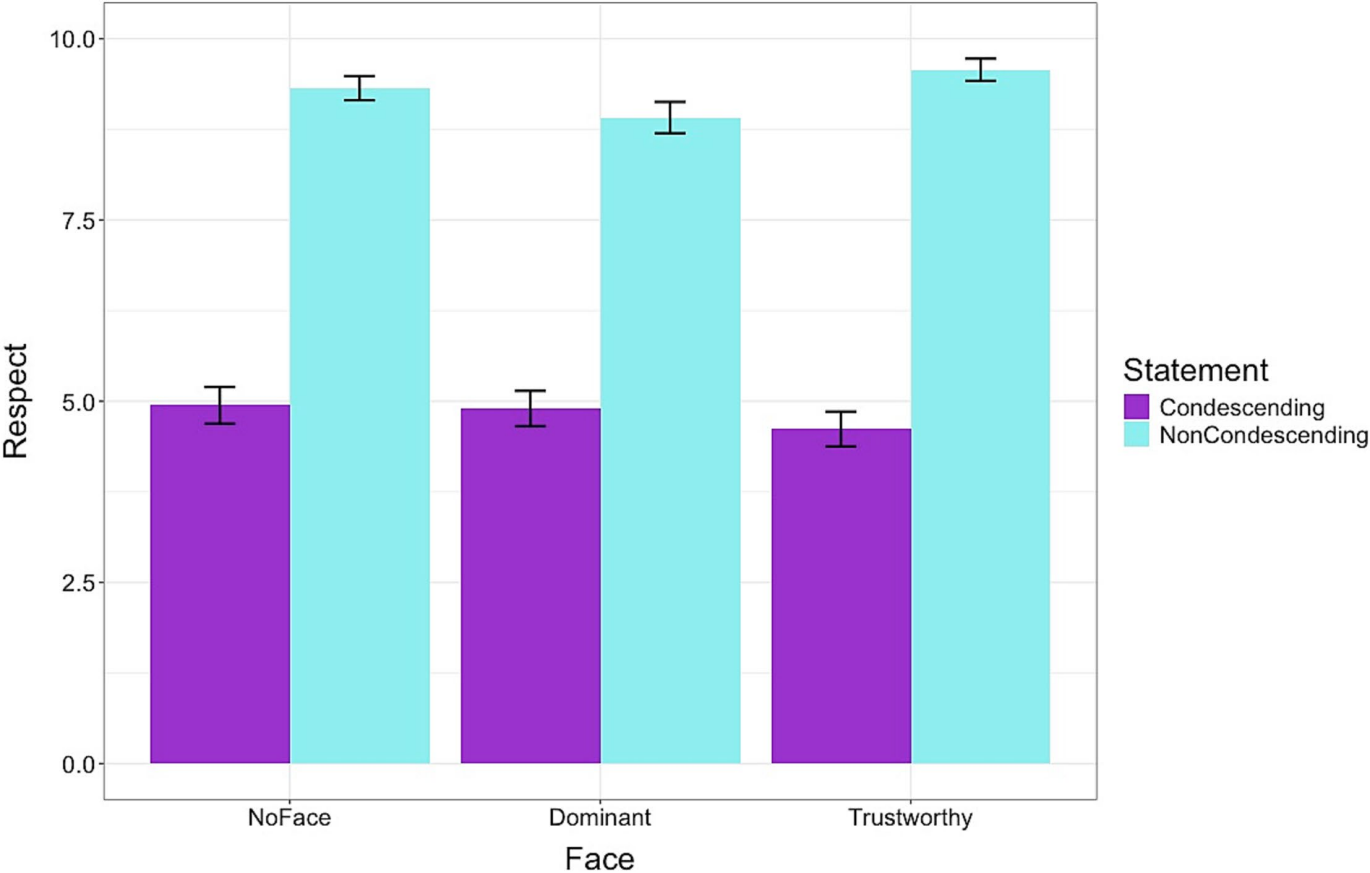
Perception of the doctor’s attitude based on facial expression and language used to provide advice. The statements designed to sound condescending reduced levels of perceived respectfulness regardless of the doctor’s look. The difference in mean respect rating was smaller for the Dominant face but larger for the Trustworthy face. Error bars represent standard errors.

For the first hypothesis (H1), results showed that condescending statements led to lower respect ratings compared to non-condescending statements across conditions [*β*: −3.05, sd: 0.26, 95% highest density interval (HDI) = −3.59, −2.58]. Post-hoc models indicated that compared to the NoFace condition, the difference in mean respect rating was smaller for the Dominant face [β: 0.28, sd: 0.10, 95% HDI = 0.08, 0.48] but larger for the Trustworthy face [β: −0.34, sd: 0.10, 95% HDI = −0.54, −0.15] (Figure 3). Similar results were obtained for the question of how trustworthy the doctor was. Across conditions, condescending statements led to lower trust ratings compared to non-condescending statements [*β*: −2.15, sd: 0.18, 95% HDI = −2.52, −1.80]. Post-hoc models showed that compared to the NoFace condition, the difference in mean trust rating was smaller for the Dominant face [*β*: 0.23, sd: 0.10, 95% HDI = 0.03, 0.42] but larger for the Trustworthy face [*β*: −0.21, sd: 0.09, 95% HDI = −0.40, −0.03].

For the second hypothesis (H2), we analyzed the preferences and perceptions of the participants. When asked which doctors they prefer, most subjects across conditions chose the doctor that made the non-condescending remark (NoFace = 89%, Dominant = 83%, Trustworthy = 85%). A chi-square test showed that differences among the conditions were not significant, *X^2^* (2, *N* = 138) = 2.4, *p* = 0.3. We found differences in the participants’ decision to pay for the doctors and travel to their office. On average, across conditions, participants chose to pay less [*β*: −2.56, sd: 0.33, 95% highest density interval (HDI) = −3.25, −1.97] and travel fewer hours [*β*: −2.66, sd: 0.30, 95% HDI = −3.28, −2.13] to visit the doctors that had made condescending remarks compared to those that had made non-condescending remarks. There was a significant interaction effect for the pay rating means. The difference between the condescending and non-condescending statements was smaller for the Dominant condition than for the NoFace condition [*β*: 0.33, sd: 0.15, 95% HDI = 0.04, 0.64] but the difference in means between the Trustworthy and the NoFace condition was not significant [*β*: −0.02, sd: 0.15, 95% HDI = −0.31, 0.28]. For travel time, there were no interaction effects.

### 3.2 Decisions

**Figure 4:**
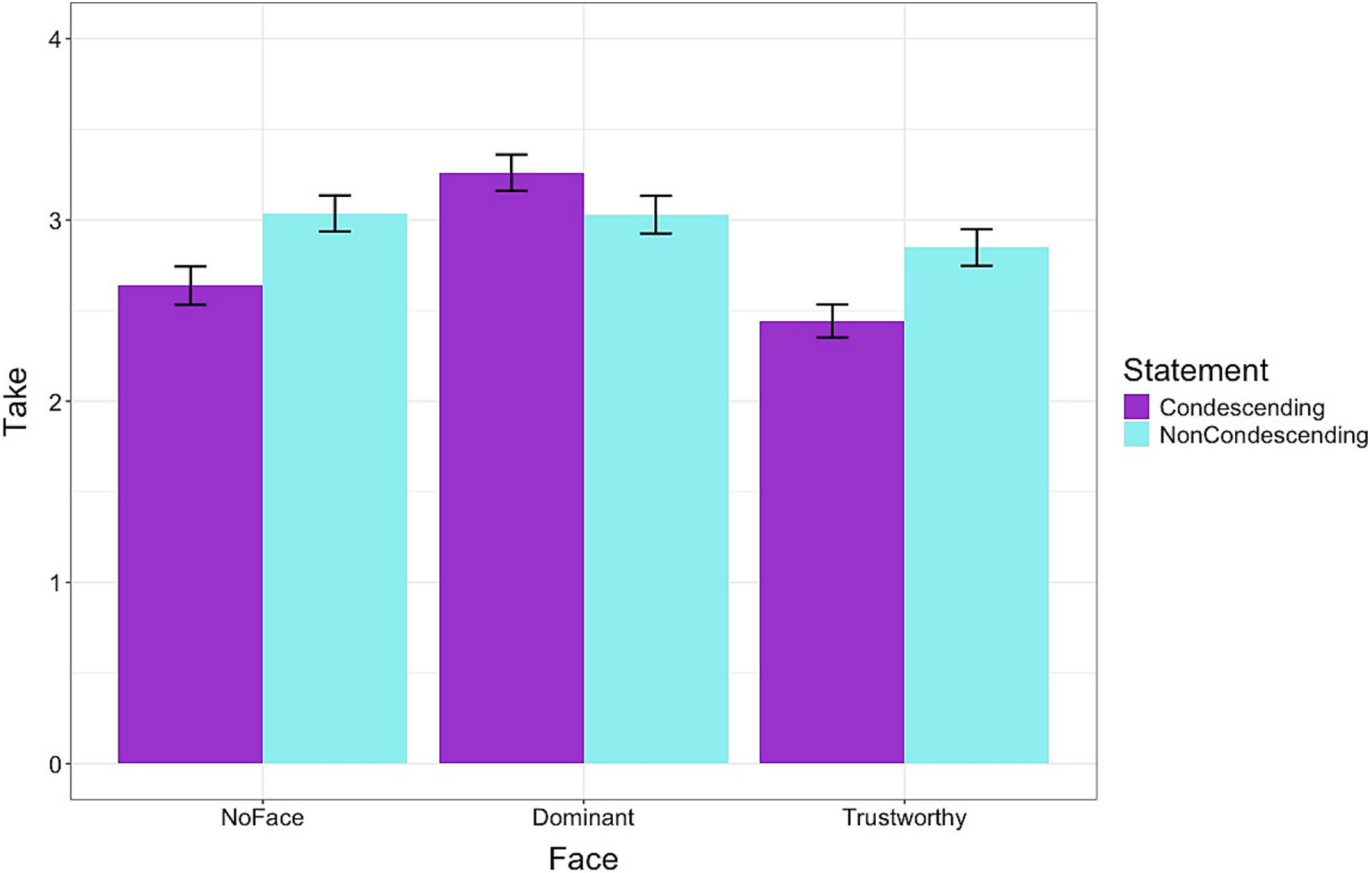
The decision to take a prescribed medication based on the doctor’s facial expression and language used to provide advice. The medication was more likely to be accepted by dominant-looking doctors than by other doctors. The mean rating was higher for the Dominant but lower for the Trustworthy condition. Error bars represent standard errors.

For the third hypothesis (H3), we analyzed the decisions that participants made about the medications prescribed by the doctors. Across conditions, no significant differences were found for the assessment of the probability that participants would experience the “Uncommon” side effect if they took the medication (means = 24.7 to 29.3), [*β*: −0.64, sd: 0.34, 95% HDI = −1.30, 0.02]. However, there was a difference in the decision to take the medication. On average, across conditions, the mean rating to take the medication was significantly lower for condescending than for non-condescending statements [*β*: −0.21, sd: 0.07, 95% HDI = −0.35, −0.07]. Post-hoc models revealed that the mean rating was higher for the Dominant [*β*: 0.58, sd: 0.11, 95% HDI = 0.37, 0.81] but lower for the Trustworthy condition [*β*: −0.50, sd: 0.10, 95% HDI = −0.69, −0.29]. There was also an interaction effect. The difference in medication take rating for the condescending statement was higher than the non-condescending statement in the NoFace condition but smaller in the Dominant condition [*β*: 0.49, sd: 0.10, 95% HDI = 0.30, 0.68]. The difference in mean rating to take the medication between the condescending and non-condescending statements was larger for the Trustworthy condition compared to the NoFace condition [*β*: −0.23, sd: 0.09, 95% HDI = −0.42, −0.05] (Figure 4).

Looking at the participants’ reasons for their decision to either take the prescribed medication or not, we found a variety of responses. Most participants chose the unlikely and the likely possibility of being afflicted by the side effect. Some chose reasons related to doctors, and a few chose reasons related to the rare side effects of the medication.

### 3.3 Physiological responses

**Figure 5:**
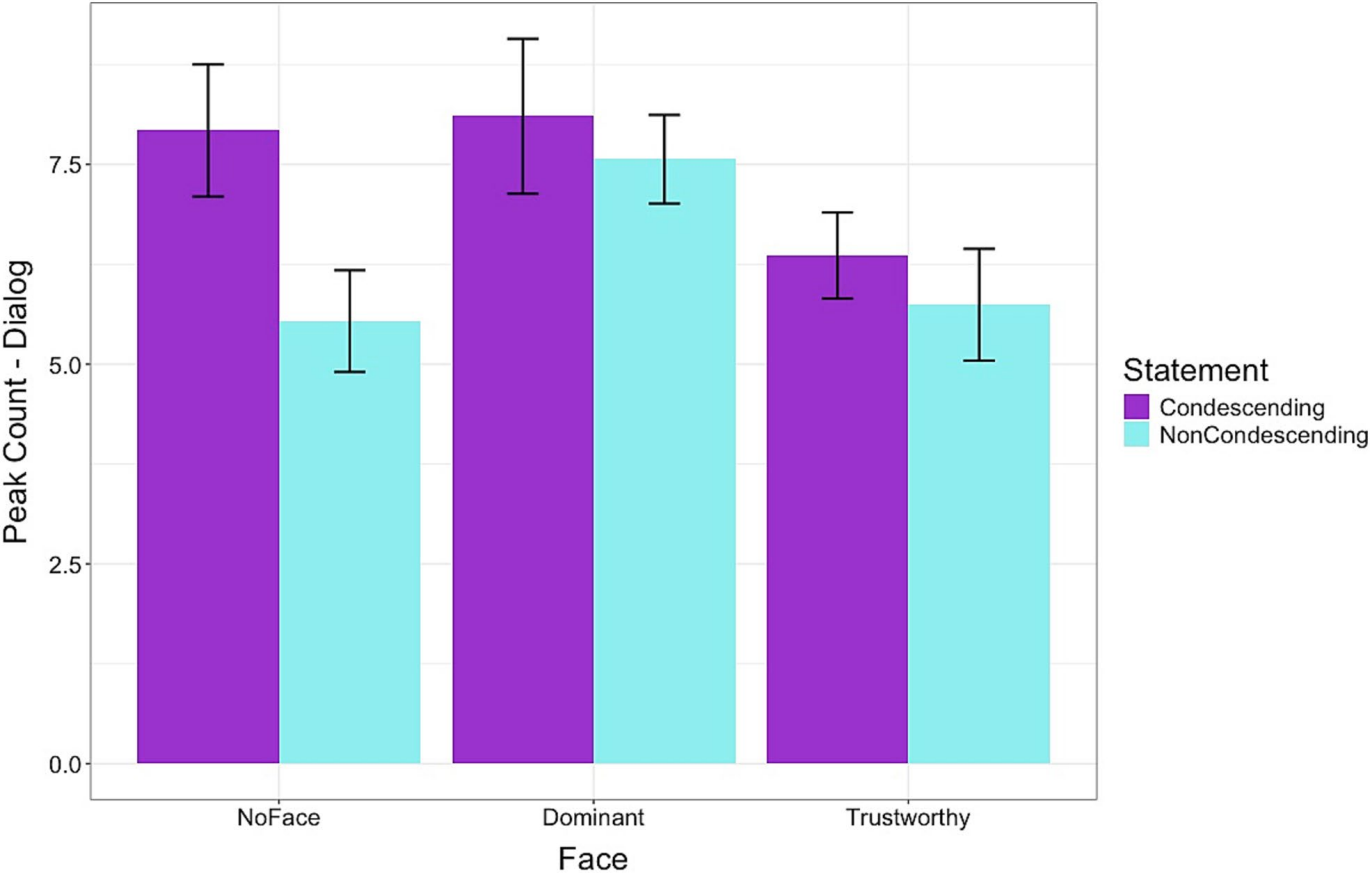
Frequency of peaks in skin conductance levels while viewing the doctor-patient dialogs. Condescending language resulted in higher arousal levels, particularly for the dominant-looking doctors. The Dominant condition led to a higher, but the Trustworthy condition led to a lower peak count than the NoFace condition. Error bars represent standard errors.

For the fourth hypothesis (H4), we analyzed the physiological responses data. Figure 5 shows the results for peak count in the Dialog segment of the survey. Across the face conditions, condescending statements resulted in higher peak counts than the non-condescending statements [*β*: 0.59, sd: 0.26, 95% HDI = 0.07, 1.12]. The Dominant condition led to a higher [*β*: 0.96, sd: 0.47, 95% HDI = 0.04, 1.88] but the Trustworthy condition led to a lower [*β*: −0.82, sd: 0.36, 95% HDI = −1.52, −0.10] peak count compared to the NoFace condition. There were no significant interaction effects indicating that the statements produced a similar result across the different conditions. For the peak amplitude of the Dialog segment, there were no significant differences for statements or conditions and no interaction effects.

**Figure 6:**
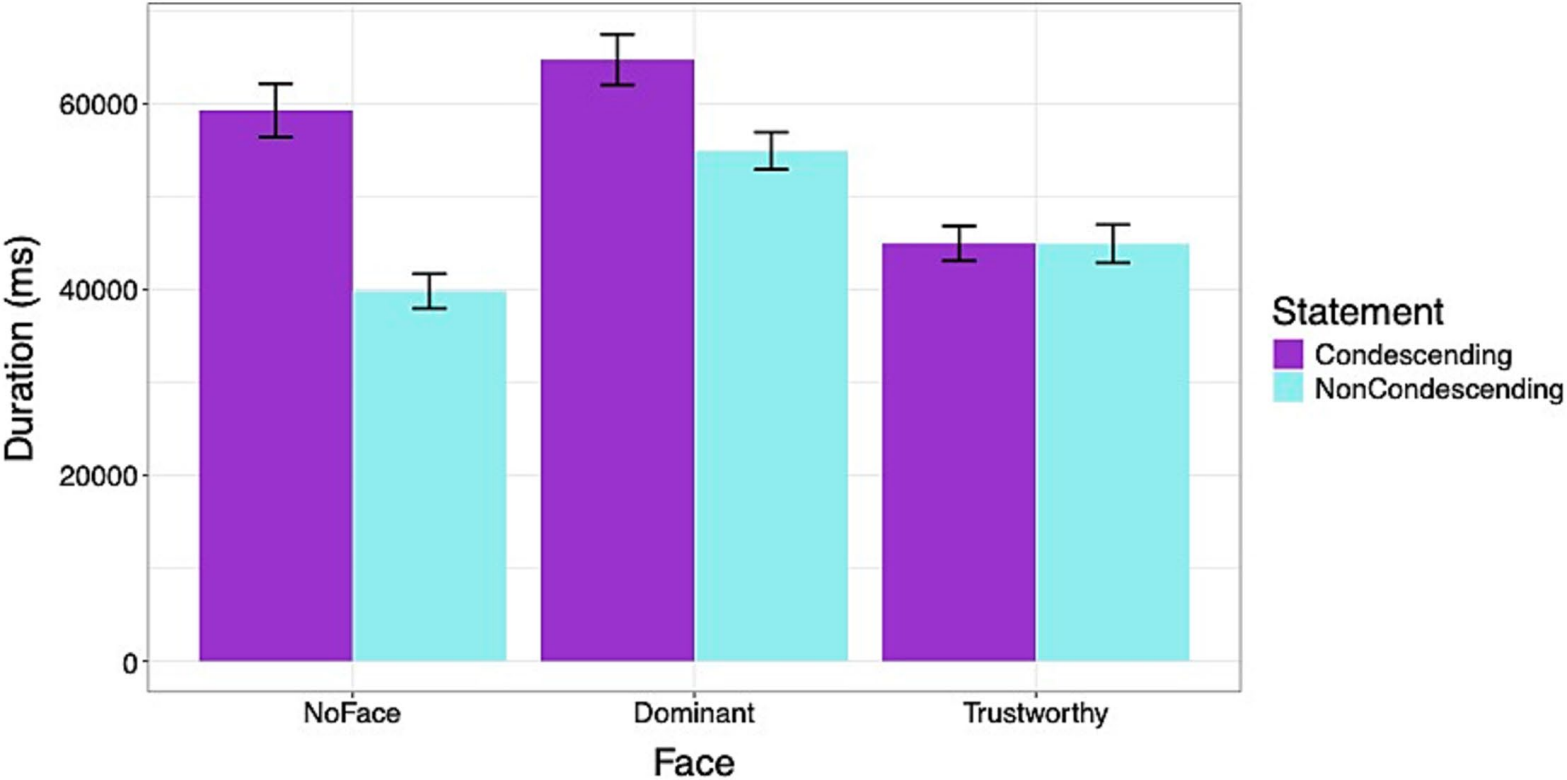
Duration of time (ms) viewing the doctor-patient dialogs. Participants spent more time viewing condescending statements, particularly when they were accompanied by no face or a dominant face. Errors bars represent standard errors.

Figure 6 shows the duration of time (ms) spent on the Dialog segment of the survey. Across the face conditions, participants spent more time viewing the condescending statements than the non-condescending statements [*β*: 4906.71, sd: 740.80, 95% HDI = 3473.91, 6359.76]. On average, across the types of statements, the Dominant face condition had a higher [*β*: 8374.53, sd: 1230.22, 95% HDI = 5976.57, 10831.16], but the Trustworthy face had a lower duration than the NoFace condition [*β*: −6522.42, sd: 1091.28, 95% HDI = −8666.58, −4367.05]. There was no significant interaction for statement type between the Dominant and NoFace conditions. However, the interaction effect was significant between the Trustworthy and NoFace conditions [*β*: −4872.01, sd: 1018.56, 95% HDI = −6920.31, −2874.12]. For both Dominant and NoFace conditions, the participants spent more time viewing the condescending statements, but for the Trustworthy condition, the participants spent an equal length of time viewing the two types of statements.

**Figure 7:**
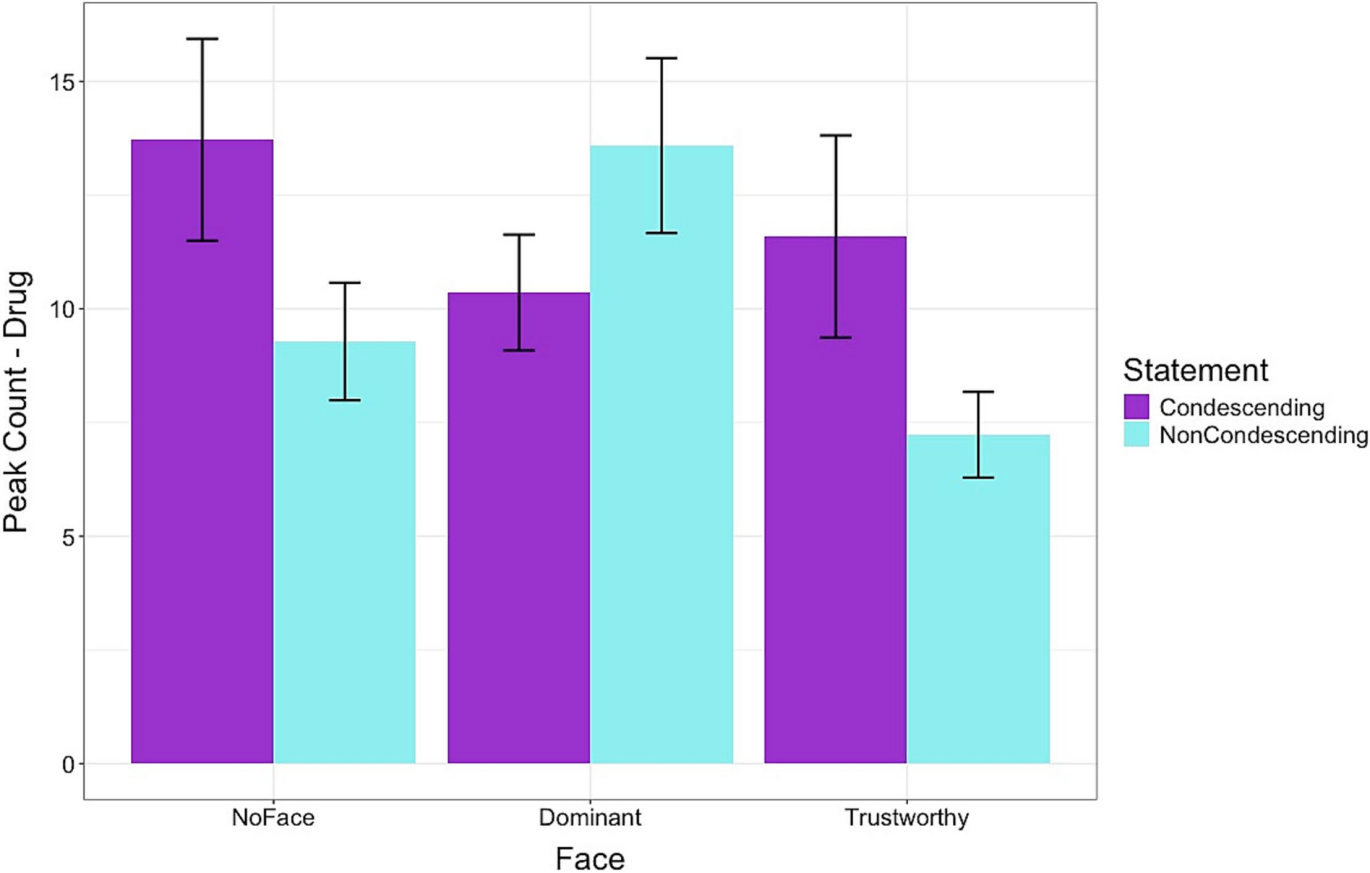
Frequency of peaks in skin conductance levels while evaluating the medication side effects and deciding to accept the prescribed medication. Condescending language resulted in lower arousal levels than non-condescending language for the dominant-looking doctors. In the Dominant condition, participants showed a lower peak count after viewing the condescending statement than the non-condescending statement Error bars represent standard errors.

Figure 7 shows the results of the Decision segment where a significant interaction effect was found for the peak count. In the NoFace condition, deciding about the medication after viewing the condescending statement resulted in a higher peak count than deciding after viewing the non-condescending statement. However, the opposite effect was found in the Dominant condition with participants showing a lower peak count after viewing the condescending statement than the non-condescending statement [*β*: −2.54, sd: 0.81, 95% HDI = −4.15, −0.95]. For the Trustworthy condition, the pattern was the same as the NoFace condition, but the differences were not significant. For the peak amplitude of the Decision segment, there was an effect for the face conditions but no interaction effects. On average, across the two statement types, the Trustworthy condition led to a higher average peak amplitude [*β*: 0.05, sd: 0.02, 95% HDI = 0.01, 0.09], but the Dominant condition did not differ across the statements.

**Figure 8:**
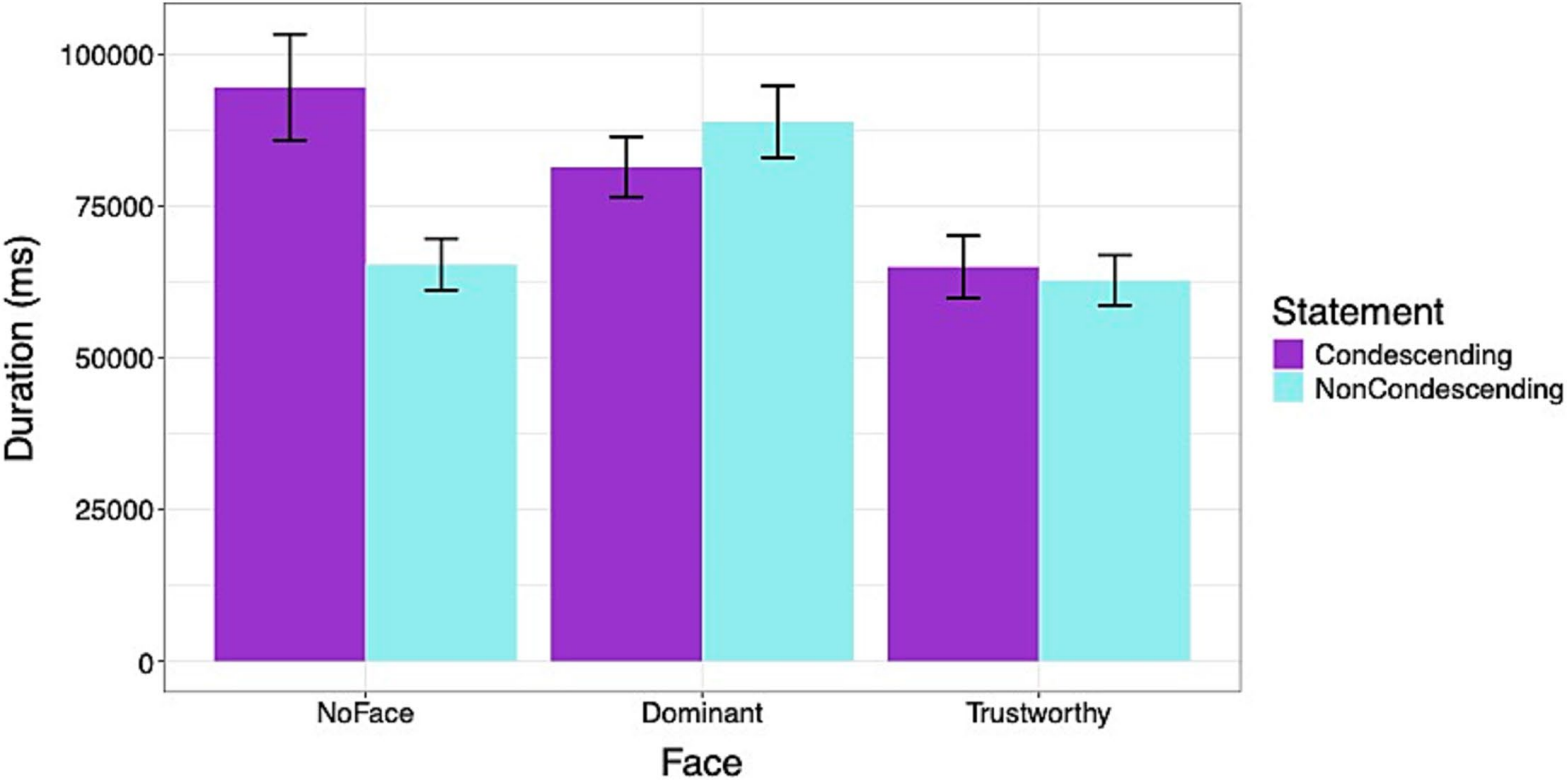
Duration of time (ms) spent evaluating and deciding to accept the prescribed medication. Participants spent more time viewing condescending statements, particularly when they were accompanied by no faces. Errors bars represent standard errors.

Figure 8 shows the duration of time spent on the Decision segment of the survey. Results showed that across the face conditions, participants spent more time viewing the condescending statements than the non-condescending statements [*β*: 4021.85, sd: 1978.28, 95% HDI = 234.20, 7934.30]. On average, across the types of statements, the Dominant face condition had a higher [*β*: 8791.90, sd: 3207.54, 95% HDI = 2573.56, 15316.60], but the Trustworthy face had a lower duration than the NoFace condition [*β*: −12429.20, sd: 2666.80, 95% HDI = −17511.69, −7229.95]. There was a significant interaction effect for the statement type between the Dominant and NoFace conditions [*β*: −7731.24, sd: 2424.60, 95% HDI = −12423.21, −2979.59] and between Trustworthy and NoFace conditions [*β*: −2907.27, sd: 2373.14, 95% HDI = −7626.72, 1543.50]. For the NoFace condition, the participants spent more time viewing the condescending statement. However, for both Dominant and Trustworthy conditions, the time spent viewing the condescending and non-condescending statements did not differ.

**Figure 9:**
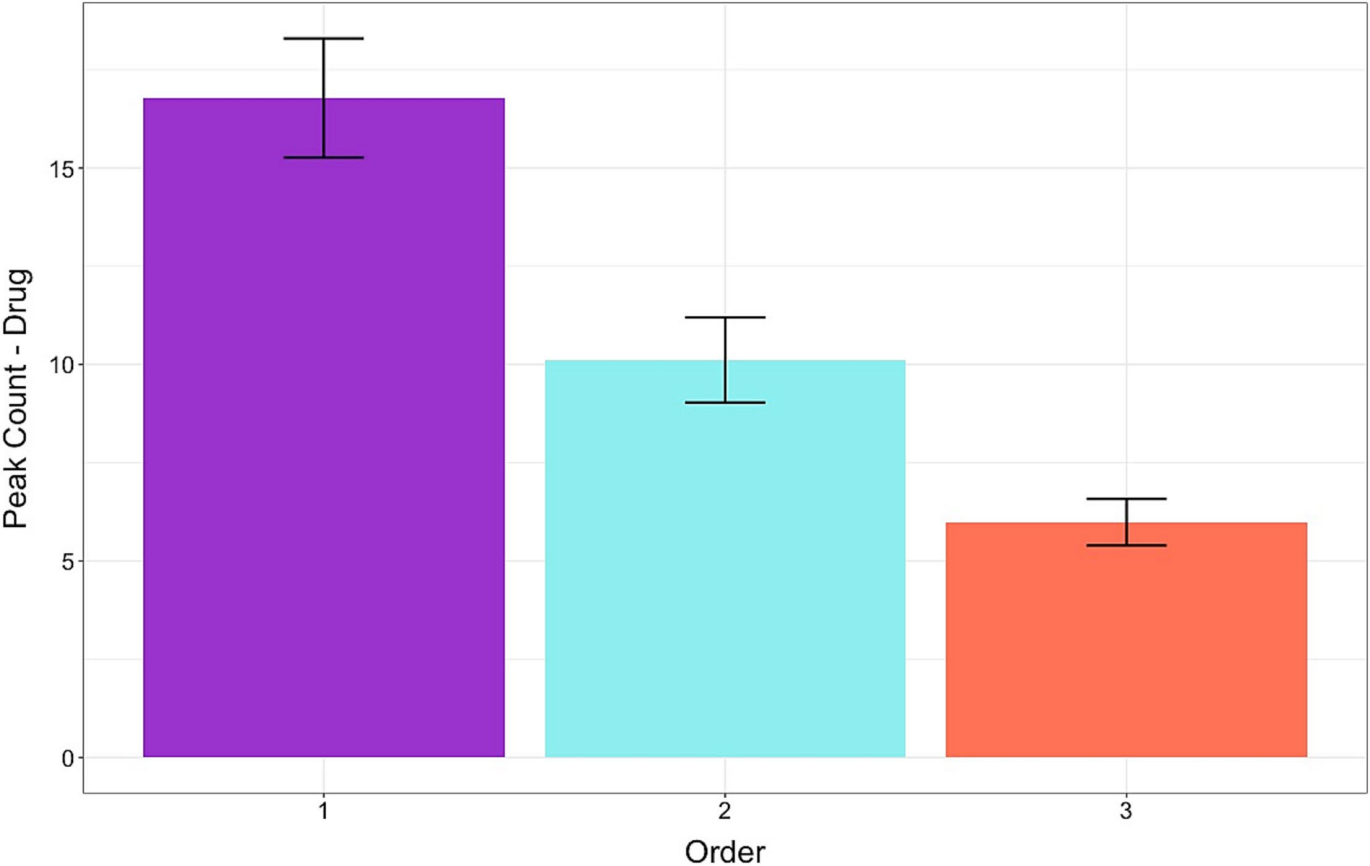
Frequency of peaks in skin conductance levels while evaluating the medication side effects and deciding to accept the prescribed medication. Arousal levels gradually decreased with repeated exposure to hypothetical doctor-patient scenarios presented in random order. Error bars represent standard errors.

The results for arousal levels following repeated exposure in a random order to the doctor-patient scenarios are depicted in Figure 9. For the dialog phase, the peak count was higher at the first exposure than at the second exposure [*β*: −1.85, sd: 0.31, 95% HDI = −2.43, −1.24]. The peak count was lower at the third exposure compared to the first and the second exposures [*β*: −1.14, sd: 0.19, 95% HDI = −1.5, −0.77]. The same results were obtained for the drug phase with a peak count higher for the first than the second exposure [*β*: −3.31, sd: 0.66, 95% HDI = −4.63, −2.03] and lower for the third compared to the first and second exposures [*β*: −2.45, sd: 0.47, 95% HDI = −3.34, −1.51].

## 4 Discussion

We investigated the perceptual and physiological responses of participants to reading the advice of a doctor who was made to use condescending remarks and look dominant. Results confirmed our H1 and H2 hypotheses showing that most participants understood the meaning of condescending and found common adverbs and adjectives added to the patient-doctor dialog for emphasis as rude and disrespectful. They found the non-condescending doctors more trustworthy than the condescending doctors and expressed their preference by choosing to pay more and travel longer distances to visit them.

For H3, we had hypothesized that the perception of being disrespected by the words of a dominant figure would stimulate anger that was previously reported to affect probability estimates and decision-making (DeSteno et al., 2000; Lerner and Keltner, 2000; Waters, 2008; Angie et al., 2011; Abel, 2023). However, the data only partially confirmed our hypothesis. In line with past research, the participants overestimated the probability of “uncommon” side effects of medication across conditions, which is estimated to be about 1% (Peters et al., 2014; Cox, 2020), but their estimates did not vary as a function of statements or images to which they were exposed. However, their decision to take those medications was impacted. Participants were less likely to accept medications from a doctor making condescending statements than from a doctor making non-condescending statements. Those effects were accentuated by facial images in opposite directions for dominant and trustworthy-looking doctors. Contrary to our expectations, participants were more likely to take medications from dominant-looking doctors than from trustworthy-looking doctors. We had hypothesized that the image of a dominant doctor giving advice would result in rejection of the medication by that doctor, but we found the opposite.

It is plausible that exposure to subtle dominance cues induced indignation rather than anger in the participant. The perceptual data showed that the participants recognized the dominant cues as offensive but results of the probability estimates and physiological reactions suggest that the offense did not stimulate negative emotions intense enough to qualify as anger. It is possible that the participants expressed their indignation toward the rude remarks of a doctor by showing their preferences without feeling very angry.

Like anger, indignation can activate the body’s stress response when one is certain that a wrongful and immoral act has been committed (Ekman et al., 1983; Landweer, 2020). However, indignation is thought to differ from anger in two dimensions: (1) the severity of the wrongful action, and (2) the object of the offense. It is posited that indignation is felt in response to a surprising or shocking act directed toward the other, whereas anger is felt in response to a grave offense directed toward the self (Drummond, 2017). For example, we may feel indignant when watching violent actions against people of a different group in a distant place on our computer screen but would feel anger if we or our kin were threatened by the same actions. Moreover, research measuring physiological reactions such as heart rate and skin conductance suggests that anger and indignation may activate the stress response differently. It has been reported that the sympathetic nervous system activity is significantly higher when anger is induced compared to other emotions (Levenson et al., 1990; Collet et al., 1997). In the present study, most participants recognized condescending cues and found the dominant doctors sufficiently offensive to shift their preference, but the experience did not vary their probability estimate as would be expected to occur with intense anger (Lerner and Keltner, 2000).

Results of our analysis to test for H4 revealed that in participants whose sympathetic nervous system was activated by the experience, arousal levels increased in response to viewing condescendingly worded dialogs. Those effects were enhanced by facial images that matched the dialog’s tone. Thus, arousal levels during the dialog phase increased when statements were attributed to a dominant-looking doctor but decreased when the same statements were attributed to a trustworthy-looking doctor. A different pattern in arousal levels emerged during the drug phase. Participants’ arousal levels decreased when making decisions to take medications from a doctor who looked dominant and sounded condescending compared to those who sounded non-condescending, suggesting that participants were calmer when accepting medication from a dominant doctor. These results agree with the idea that the brain uses arousal levels as information and the levels may be task-dependent (Storbeck and Clore, 2008; Yoneda and Morita, 2021).

Our results correspond with past research showing that words and faces with emotional meaning elicit different pattern of arousal and are processed differently in the brain (Rellecke et al., 2011; Juuse et al., 2024). Our participants spent more time viewing condescending words and had higher arousal levels regardless of the accompanying images. However, the presence of images modulated their response in the direction of the emotion depicted, with a dominant face enhancing and a trustworthy face diminishing their response. Additionally, we observed a similar pattern of decline in arousal levels with repeated exposure to emotional stimuli as previously reported (Juuse et al., 2024). The data are in line with theories about habituation that explain the brain’s need to conserve energy (Peters et al., 2017).

The hypothetical scenario induced arousal levels high enough to be detected in only some participants even though most found the condescending remarks offensive. The lack of a stress response in most participants indicates a low level of emotional reactivity in accordance with surprise or shock. It is also possible that those who showed a detectable response were able to imagine themselves as the patient who was offended and were shocked or moderately angry, whereas others could not put themselves in place of the patient against whom the wrongful act was perpetrated. Past research has shown that individuals vary in creating vivid and negative images of risk consequences that affect their judgment and physiological reactions to stress stimuli (Traczyk et al., 2015; Beevor et al., 2024).

Although probability estimates were not affected by exposing participants to dominance cues, decision-making was impacted. Participants stated that they were more likely to take the prescribed medication from a condescending and dominant-looking doctor than from non-condescending or trustworthy-looking doctors. Contrary to our hypothesis, the arousal level of the participants who showed a stress response was lower when evaluating the medication and considering accepting the drug from an offensive doctor than a non-offensive doctor. These data agree with findings that authority figures such as doctors can secure compliance by using commanding language that is not intimidating enough to stimulate anger or fear (Hansia et al., 2023). We think that by simulating a doctor-patient dialog and introducing subtle dominance cues, we created a condition for compliance and lowering the participant’s stress levels. Participants were indignant with the rude comments of the condescending doctor but not sufficiently angry with the doctor as an authority figure to reject her prescribed medication. This hypothesis is also supported by the comments participants made when asked to explain their preference for the doctors. Some stated that while they considered the doctor’s comments as rude, they found her to be clear and knowledgeable. Whether patients in real life agree with a dominant doctor or comply with her advice may depend on the intensity and type of emotions that the doctor’s verbal and non-verbal cues stimulate in the patient (Ambady et al., 2002a; Ahmed and Bates, 2016; Liu et al., 2022). Moreover, patients with low confidence levels or low tolerance for uncertainty might be more compliant and accepting of a dominant doctor (Gudjonsson and Sigurdsson, 2003).

### 4.1 Limitations and future directions

A possible confound that could have affected the results is the adverse events of the medication that each doctor in our hypothetical scenarios offered the participants. As the drug offered by each doctor was designed to be slightly different in terms of side-effects, we cannot rule out the possibility that the drugs influenced the participants’ responses. However, given that all participants were exposed to the various types of medications and the reasons they gave for taking the medication was so varied, we do not think that the drug’s side-effects created a systematic bias in the data. A second potential limitation is the reduced sample size for SCR analysis that we chose to use. Given that we were attempting to measure autonomic responses to multiple sets of doctor-patient scenarios, we had many participants that did not have a detectable stimulus-elicited SCR for all the scenarios. Individual differences in electrodermal activity are not uncommon and could be due to demographic variables such as culture and ethnicity (Boucsein et al., 2012). In line with previous research (Wilhelm and Roth, 1996), we removed the non-responders in our study, focused on a subset of participants whose SCR levels reached the threshold set by the iMotions program (Farnsworth, 2019), and reported the data that were not included in the final analysis. Nonetheless, as the final sample size for the SCR analysis was significantly smaller than the sample size originally planned, the reported effects should be interpreted with caution and replicated in future studies.

Despite the limitations, the current study has opened several paths for further investigation. One issue is people’s physical attributes. Our simulated patient participants were mostly female, who were asked to evaluate and decide based on images of young female doctors who were either dominant or trustworthy looking. Past research has shown that men and women differ in emotion-induced probability assessments, with males having less pessimistic risk estimates than females (Lerner et al., 2003). Also, differences have been found in how patients respond to linguistic cues from female versus male doctors. In online reviews, female physicians were perceived to have more positive traits such as warmth and personable skills than their male counterparts (Gupta and Jordan, 2024). Furthermore, patients’ perception of doctors’ nonverbal cues during communication was found to vary by the doctor’s gender (Mast, 2007). Hence, it is important to look at the gender of both the participants and the doctors when testing the effect of subtle dominance cues on probability assessment and decision-making. The doctor’s age, perceived attractiveness, and ethnicity could also be factors to consider in future research as previous studies have shown that facial images of older and less attractive adults impact the perceived trustworthiness of the depicted individual (Li et al., 2022), and people are judged differently for dominance and affiliation based on their ethnicity as displayed in a facial image (Hess et al., 2000).

A second issue is how the brain activates emotions in response to dominant cues. Although condescending speech in written text can be easily detected by Natural Language Processing (Wang and Potts, 2019; Mendelsohn et al., 2020), people’s emotions may not be as intensely activated when they read the text as when they see a dominant face or hear a person talk with a condescending tone of voice. Demeaning verbal and nonverbal cues affect patient’s emotions (Ambady et al., 2002a, 2002b; Haskard et al., 2008; Rule et al., 2012), but they may do so much faster and with greater intensity if voices are heard in combination with viewing animated facial expressions (Schirmer and Adolphs, 2017). Another factor that could influence the intensity of emotions is the ability to imagine implicit aggression inflicted toward oneself or others. Individual differences have been reported in people’s ability to summon vivid images of emotional situations (Beevor et al., 2024) and the strength of invoking those mental images has been linked to risk perception and physiological responses to emotional events (Traczyk et al., 2015). Further research is required to determine if the outcome of hypothetical scenarios such as the one used in the current study is dependent on participants’ ability or willingness to imagine the situation with sufficient intensity to set in motion a detectable bodily response.

## 5 Conclusion

We showed that subtle dominance cues in text can be easily detected, but contrary to our predictions, they increased compliance and reduced the stress response. The study contributes to the burgeoning research on the use of condescending language in social media and other online sources (Bacile et al., 2018; Ortiz, 2022). Understanding the impact of speech by dominant figures on influencing people’s risk assessment and decisions related to health or policy requires further examination of how the brain and body may react to verbal and nonverbal cues that are used consciously or subconsciously to express a dominant attitude. The study aids in understanding how emotions during social interactions may be used by the brain as information to channel its energy resources adaptively and facilitate decision-making. In the age of growing telemedicine, where social interactions between patients and healthcare providers or chatbots occur at a distance, (Adeghe et al., 2024) knowing how patients’ emotions guide their perceptions and choices will be important in designing better programs to manage care.

## Data Availability

The datasets presented in this study can be found in online repositories. The names of the repository/repositories and accession number(s) can be found below: Open Science Frame-work (OSF) https://osf.io/23bhj/?view_only=b9e0db97a69d40aeabfeb0aa60bad295.
